# The associations of stress, pleasure and emotion to voice‐hearing: An ecological momentary assessment study

**DOI:** 10.1111/papt.12598

**Published:** 2025-05-19

**Authors:** Kelly Cusworth, Sharla Cartner, Georgie Paulik, Neil Thomas, Guillermo Campitelli, Danielle C. Mathersul

**Affiliations:** ^1^ School of Psychology Murdoch University Perth Western Australia Australia; ^2^ Perth Voices Clinic Perth Western Australia Australia; ^3^ Centre for Mental Health Swinburne University of Technology Melbourne Victoria Australia; ^4^ School of Population Health Curtin University Perth Western Australia Australia; ^5^ Precision Medicine Centre, Health Futures Institute Murdoch University Perth Western Australia Australia; ^6^ War Related Illness and Injury Study Center (WRIISC) Veterans Affairs Palo Alto Health Care System Palo Alto California USA

**Keywords:** auditory verbal hallucinations, ecological momentary assessment, emotion, reactivity, stress, voice‐hearing

## Abstract

**Background:**

Negative emotions and stress are theorised to play a role in the onset and maintenance of voice‐hearing experiences. However, previous research has not explored these temporal relationships in daily life using differentiated psychological constructs.

**Aim:**

Using ecological momentary assessment, this study examined the moment‐to‐moment relationships between negative and positive emotion valence and intensity, stressful and pleasurable events, and voice‐hearing onset.

**Materials & Methods:**

Forty voice‐hearers completed seven days of smartphone‐based surveys, rating their emotions and their intensity, perceived stress and pleasure of life events, and presence of voice‐hearing.

**Results:**

Multilevel modelling showed that stressful events, but not pleasurable events, were significantly predictive of voice‐hearing, both concurrently and in the next time point. Neither negative nor positive emotion intensity predicted voice‐hearing, nor did they moderate the relationship between voice‐hearing onset and stressful or pleasurable events, respectively.

**Discussion:**

These findings suggest that factors which differentiate perception of stressful events from self‐reported negative emotions may be useful intervention targets, such as mitigating prolonged external stressors, reducing sensitivity to external stressors and targeting negative perceptions or resistance to these stressors.

**Conclusion:**

Clinically, our findings underscore the relevance of stress and a negative perception of externally oriented events, with further research needed to explore useful interventions for targeting these mechanisms.

## BACKGROUND

The experience of hearing voices (“auditory verbal hallucinations”) is a transdiagnostic phenomenon that can arise within various mental health conditions, including schizophrenia, bipolar disorder and post‐traumatic stress disorder, as well as amongst non‐clinical populations (e.g. behavioural strategies, medication de Leede‐Smith & Barkus, 2013; Larøi, 2012; Waters & Fernyhough, 2017). Involving hearing the voice of someone or something that is not physically present, voice‐hearing can be distressing and can significantly impact an individual's quality of life (de Leede‐Smith & Barkus, 2013; Upthegrove et al., 2016). Understanding the factors contributing to the occurrence and maintenance of voice‐hearing in daily life is important for developing effective interventions.

Theoretical models of voice‐hearing (Waters et al., 2012) suggest it arises from an interaction between abnormal bottom‐up processes (i.e. neural activation patterns produce hypersalient auditory signals) and top‐down processes that influence how sensory experiences are interpreted. Affective experiences, including emotion, stress and mood, can play a prominent role at all levels of this hierarchy; however, existing voice‐hearing research is confounded by a lack of differentiation between these affective processes.

Emotions, particularly the tendency for intense and long‐lasting emotional responses, and maladaptive attempts to regulate this, are theorised to disrupt top‐down inhibitory control mechanisms, lowering the threshold for error to occur and resulting in mental activity being misattributed as externally generated and/or threatening voice‐hearing experiences (Mertin & O'Brien, 2013; Thewissen et al., 2011). Voice‐hearers often report feeling a range of negative emotions, such as fear, anger and shame, both during and outside of voice‐hearing experiences (Bortolon et al., 2021; Castiajo & Pinheiro, 2021; Hugdahl, 2015; McCarthy‐Jones, 2017). However, the directionality of this relationship remains unclear. Some studies suggest that negative emotions may trigger or exacerbate voices, while others suggest that voices may lead to negative emotions (Rosen et al., 2018; So et al., 2021). It is most likely that this relationship is bi‐directional, with negative emotion potentially triggering voice‐hearing and, in turn, voice‐hearing intensifying negative emotion, creating a self‐reinforcing cycle (So et al., 2021).

While emotions refer to more specific responses and are typically characterised by their subjective quality, rapid onset and often brief duration, stress is conceptualised as a stereotyped response to negative situational demands or external threats, involving a more prolonged physiological and psychological activation (Gross & Ford, 2023). While stressful events can elicit specific emotions (e.g. frustration or anger), stress also involves an evaluation or belief about one's ability to cope with those situational demands. According to Gross' Extended Process Model of Emotion Regulation (2015), stress can trigger emotion regulation processes, as individuals attempt to manage the emotional and physiological impact of the stressors (Gross & Ford, 2023; Sheppes et al., 2015). In psychosis, a population with a high occurrence of voice‐hearing, clinical populations show heightened stress reactivity when compared to controls (Myin‐Germeys et al., 2001, 2003; Paetzold et al., 2021) and increased stress sensitivity predicts more frequent and severe voice‐hearing experiences (DeVylder & Hilimire, 2015). This suggests that heightened physiological and psychological responses to everyday stressors may lower the threshold for the emergence or intensification of voices, though the precise relationships between these mechanisms underlying this relationship are unclear.

Stress sensitivity has been conceptualised as increased negative emotion in response to minor stresses in daily life, and theories propose the effects of stress on psychotic experiences such as voice‐hearing are at least partly mediated through experiences of negative emotion (Garety et al., 2007; Klippel et al., 2022; Myin‐Germeys & van Os, 2007). However, to our knowledge, no studies have specifically explored the temporal inter‐relationships between stress, negative emotion and voice‐hearing in daily life.

Additionally, while stressful events and negative emotions have received some investigation, the role of positive emotions and pleasurable events is less explored. Some studies suggest voice‐hearers have lower levels of positive emotion (Cho et al., 2017; Martins et al., 2019; Oorschot et al., 2012), while others have found little difference in self‐reported experiences of pleasurable emotional states (Cohen & Minor, 2010). Several theories may implicate positive emotion, particularly intensive emotion, in voice‐hearing onset: (1) if voice‐hearers tend towards hyperarousal and negative reactivity, they may be more likely to view similar physiological sensations such as intense positive emotional experiences as aversive (Williams et al., 1997); (2) positive emotion arousal may also activate neural networks involved in voice‐hearing (Gross & Jazaieri, 2014; Hoffman & McGlashan, 2001); and (3) intense positive emotions might lead to increased self‐focused attention, where voice‐hearers become more aware of their internal thoughts, feelings and bodily sensations, which has been associated with increased voice‐hearing (Bates et al., 2015; Bell et al., 2024). To our knowledge, no studies have explored the relationship between pleasurable event ratings and voice‐hearing in daily life. Therefore, understanding the dynamics of positive emotions in voice‐hearers is essential for developing comprehensive interventions and support strategies addressing both negative emotional experiences associated with voice‐hearing and enhancement of positive emotional well‐being.

### The present study

To clarify the relationships between stress, emotion and voice‐hearing, this study will examine their temporal inter‐relationships during daily life using an ecological momentary assessment (EMA) approach. EMA allows for examination of the dynamic, time‐varying interplay between variables, providing a more nuanced understanding of voice‐hearing predictors in daily life than traditional retrospective self‐report measures. To examine the potential role of both positively and negatively valenced events and emotions, both will be examined.

We take a transdiagnostic approach exploring voice‐hearing as a dimensional psychopathology experience that cuts across diagnostic boundaries to explore novel insights and better understand and support those experiencing voice‐hearing.

To investigate these relationships, we proposed the following hypotheses:

1. The intensity of negative emotions will predict the presence of voice‐hearing.
2. The intensity of positive emotions will predict the presence of voice‐hearing.
3. The intensity of stressful events will predict the presence of voice‐hearing.
4. The intensity of pleasurable events will predict the presence of voice‐hearing.
5. This relationship between stressful events and voice‐hearing will be moderated by negative emotion intensity.
6. This relationship between pleasurable events and voice‐hearing will be moderated by positive emotion intensity.

## METHOD

### Participants

A community sample of 44 voice‐hearers completed the research for $50 remuneration. Participants were recruited via Perth Voices Clinic, a Psychology Clinic Research Registry, and the Hearing Voices Network. Subjects provided written informed consent for a protocol approved by the Murdoch University Human Research Ethics Committee (Approval number 2022/019).

#### Inclusion and exclusion criteria

All participants were required to be over the age of 18 years and able to consent to participate. They were required to own and be proficient using a smartphone (that was not an Oppo or Huawei, due to incompatibility with app system requirements) and have access to internet connection periodically for installation of the app and periodic upload of completed data. Participants were required to be experiencing frequent and persistent voice‐hearing (present for more than 1 month, occurring at least once a week) as assessed using the MINI 7.02, Psychotic Disorders Scale (questions K6; Sheehan et al., 1998) and cross‐checked with their response on the Hamilton Program for Schizophrenia Voices Questionnaire (HPSVQ; Kim et al., 2010) to the item, “During the past week, how frequently did you hear a voice or voices?” (rated on an ordinal 0–4 scale; No Voices, Less than Once a Day, Once or Twice a Day, Several Times per Day, All the Time). The HPSVQ is a brief 9‐item self‐report questionnaire providing a multidimensional measure of voice‐hearing.

Participants were also systematically assessed by the interviewer and excluded for the presence of co‐occurring delusions of reference, persecution, or grandeur, using questions derived from the MINI 7.02 Psychotic Disorders scale (Sheehan et al., 1998).

### Materials

#### Demographics

Participant demographics were collected, including age, gender, ethnicity, highest level of education, current psychiatric medication, any current diagnoses and age of onset of voice‐hearing.

#### Ecological momentary assessment (EMA)

This EMA study used SEMA^3^ (Smartphone Ecological Momentary Assessment), a software app designed for intensive longitudinal research using iOS or Android smartphones (O'Brien et al., 2024). Before recruiting participants, a lived experience panel of voice‐hearers was consulted to review EMA items and provide feedback on item appropriateness, their understanding of the questions, participant burden, and any potential risks or foreseeable issues. EMA items not analysed in this paper are not reported in this paper.

##### Voice‐hearing onset

Participants were asked, “Since the last notification, have you heard voices that other people cannot hear?”, with Yes or No response options. Participants were also asked to provide ratings of distress and dysfunction relating to their voices (not analysed in this study).

##### Stressful and pleasurable life event ratings

Participants were asked to rate their perception of their experience using a slider scale from 0 (no stress/pleasure) to 100 (high stress/pleasure). One question related to stress and asked participants to, “Move the slider to indicate how *stressful* your life has been for you since the last notification”. One question related to pleasantness and asked participants to, “Move the slider to indicate how *pleasant* your life has been for you since the last notification”.

##### Emotion

Participants were asked to select the strongest emotion felt since the last notification, using the options *Happy, Enthusiastic, Confident, Alert, Relaxed, Sad, Irritable, Nervous, Ashamed, Sluggish*. Participants also had the option to select *Neutral*, “I felt another emotion more strongly (please specify)”, or “I can't identify what I have been feeling”. These 10 emotions were adapted from the Positive and Negative Affect Schedule – Expanded Form (PANAS‐X; Watson & Clark, 1994), a self‐report measure assessing the extent to which participants have experienced various emotions during a specified time frame. The PANAS‐X includes the 20 items from the original PANAS, which measure two broad dimensions of affect (positive and negative), and 40 items assessing 11 specific emotional states: fear, sadness, guilt, hostility, shyness, fatigue, surprise, joviality, self‐assurance, attentiveness and serenity. The PANAS‐X has demonstrated strong psychometric properties, including high internal consistency (*α* = .83–.90 for negative affect; .84–.91 for positive affect), test–retest reliability (.73 for negative; .76 for positive) and construct validity (Watson & Clark, 1994).

##### Emotion intensity

After choosing one emotion, participants were asked to rate how intensely they had felt this emotion using a slider scale of 0 (no intensity) to 100 (extreme intensity).

### Procedure

After an initial eligibility interview, participants completed a battery of questionnaires as part of another study (not reported here) either online (via Qualtrics) or via direct mail. The questionnaires took approximately 30–40 min. Following this, participants were contacted, either in person or via phone call, and provided instructions and training for participating in this EMA study. This included step‐by‐step guidance in setting up the SEMA^3^ application (O'Brien et al., 2024) on their personal Android or iOS smartphone; technical troubleshooting to ensure appropriate receipt of notifications; tailoring EMA survey schedules to the participant's preferred start date and typical daily wake‐up time; and guidance on how to complete a questionnaire once received. A check‐in text message and/or phone call was provided to every participant within the first 24 hours, and participants were encouraged to contact the researcher for technical support at any time. Participants completed an EMA survey (minimum 12‐item, maximum 18‐item depending on response) over 7 days, with 10 prompts per day, occurring at stratified pseudorandomised intervals of 90‐min blocks with a minimum of 30 min between notifications, within a 12‐h window programmed to the participants' individualised sleeping habits. Assessments not completed within 30 min were logged as missed. Participants also received one additional notification per day asking two questionnaires related to sleep to be completed at any time on that day.

### Data analysis

EMA data were analysed using multilevel modelling in RStudio (Team, 2020) with the lme4 (Bates et al., 2015) and lmerTest packages (Kuznetsova et al., 2017). Predictors were group‐mean‐centred to control for between‐person differences (Paccagnella, 2006). Due to the large differences in the measurement of some predictors and the dependent variable, all predictors measured on a scale from 0 to 100 were rescaled to 0–10 for meaningful interpretation and model stability (Hosmer et al., 2013). Logistic random effects models were conducted using family binomial (glmer). The default optimiser, Nelder Mead, was applied; however, some models were fitted with the bobyqa optimiser when Nelder Mead models failed to converge.

According to the PANAS‐X, the Sluggish and Relaxed emotions load onto the Fatigue and Serenity subscales respectively, and not consistently onto either Positive or Negative Affect higher‐order factors. Furthermore, the authors of this paper considered the possibility that endorsement of intensive Sluggish and Relaxed experiences may also indicate the absence of emotion intensity or disturbance, in line with valence‐arousal models of affect (Heller, 1993; Watson & Tellegen, 1985), and therefore potentially confound the findings. To ensure rigour and completeness in this study's findings, sensitivity analyses were conducted that removed Sluggish and Relaxed from Negative and Positive Emotion Intensity totals, respectively, for Hypotheses 1, 2, 5 and 6. In the Results, the analyses with the total emotion intensity variables are first listed for that hypothesis, followed by those with the emotion intensity values minus Sluggish or Relaxed respectively.

For Hypotheses [Link], [Link], [Link], [Link], both concurrent and time‐lagged (dependent variable Voices *t* − 1) were conducted. For Hypotheses 5 & 6, the Independent Variable (Stress/Pleasurable Event Ratings) and Moderator Variable (Negative/Positive Emotion Intensity) were in the preceding time point (*t−*1) to the Presence of Voice‐hearing.

The odds ratio was calculated using broom.mixed package (Bolker & Robinson, 2024). During analysis, assumptions of linearity, normality and homoscedasticity were violated, and many models had issues with convergence and singularity. As bootstrapping lowers standard error and improves sampling variability (McNeish & Stapleton, 2014), these violations and issues were overcome by bootstrapping the confidence intervals (5000 times) at the 95% level on the odds ratio statistic. Significant testing was determined by *p*‐values < .05.

## RESULTS

### Data cleaning and preparation

Three participants were excluded due to technical issues in receiving and completing their survey notifications, and one participant was excluded due to missing data on all items required for analysis. Thus, the final sample comprised 40 participants.

Three‐level models (Observations = 1, Day = level 2, Participants = level 3) were compared to two‐level models (Observations = 1, Participants = level 2) using ANOVA to detect which model had lower AIC and BIC fit indices (Burnham & Anderson, 2004). The two‐level models indicated a better fit of the data with lower fit indices, and there were no significant differences between the two‐ and three‐level models. Therefore, two‐level models were most parsimonious and had stronger statistical power (Tables A and B in Appendix S1).

### Sample demographics and descriptives

Basic demographics and clinical characteristics of the final sample are summarised in Table 1.

**TABLE 1:** Sample demographics.

Age, *M* (*SD*)	35.15 (11.71)
Age of voice‐hearing onset, *M* (*SD*)	18.27 (13.56)
Gender	
Female or woman	26 (65%)
Non‐binary or genderqueer or third gender	1 (2.5%)
Male or man	13 (32.5%)
Employment	
Employed	15 (37.5%)
Unemployed	16 (40%)
Student	6 (15%)
Other (e.g. retired, volunteer)	3 (7.5%)
Marital status	
Single	24 (60%)
Married or de‐facto or partnered	10 (25%)
Separated/divorced/widowed	6 (15%)
Education	
High school	17 (42.5%)
Trade/technical/vocational training	13 (32.5%)
Bachelor's degree	8 (20%)
Master's degree	2 (5%)
Current or past diagnosis	
Psychotic disorder only	10 (25%)
Co‐occurring psychotic & non‐psychotic disorder	10 (25%)
Non‐psychotic disorder only	13 (32.5%)
No diagnosis	2 (5%)
Unclear/unknown	5 (12.5%)
Current medications^a^	
No MH medication	8
Antipsychotics^b^	28
Mood stabilisers	5
Antidepressants	19
Benzodiazepines	7
Other sleep‐related medications	1
Stimulant medications	3

^a^ Some participants endorsed more than 1 category.

^b^ Due to the prevalence of antipsychotic medication use in the sample, an independent samples *t*‐test was conducted to explore differences in emotion intensity between voice‐hearers with antipsychotic medication use (*n* = 28, *M* = 66.40, *SD* = 11.53) and voice‐hearers without antipsychotic medication use (*n* = 12, *M* = 62.52, *SD* = 15.98). There was no statistically significant difference between the two groups, *t*(38) = −0.75, *p* = .46.

Completion rates showed 1775 (63.34%) completed surveys across 2800 time points.^1^ The mean number of prompts answered by participants was 44.38 (*SD* = 14.54) out of 70 prompts, with a response rate range from 22.86% to 98.57%. As there were only two non‐consecutive days where the participant made no response and no obvious response bias, the data were retained as per guidelines by Bell et al. (2023). Voices were reported in 74% (*n* = 1321) time points.

Table 2 shows descriptive data of the analysed variables. Additional results can be found in Supporting Information (Figures A and B in Appendix S1).

The intensity of negative emotions will predict the presence of voice‐hearing.

**TABLE 2:** Descriptive statistics for event pleasantness and stress, and positive and negative emotion intensity.

Event ratings	Time points (*N* = 1775)	Time points with voice‐hearing (*n* = 1321)	*M*	*SD*
Pleasurable event ratings			62.28	21.91
Stressful event ratings			41.33	27.28
Emotion intensity				
Positive emotion	716	515 (72%)	69.04	17.12
Positive emotion (relaxed removed)	444	325 (73%)	72.4	16.03
Negative emotion	642	531 (83%)	68.16	21.08
Negative emotion (sluggish removed)	440	400 (91%)	69.87	21.89
Happy	172	114 (66%)	75.82	14.69
Enthusiastic	53	35 (66%)	71.09	14.98
Confident	64	53 (83%)	64.28	17.42
Alert	155	123 (79%)	63.95	16.98
Relaxed	272	188 (69%)	68.37	17.59
Sad	142	114 (80%)	67.22	27.91
Irritable/angry	117	105 (90%)	66.98	19.18
Nervous	156	150 (96%)	73.85	17.53
Ashamed	25	23 (92%)	73.56	14.96
Sluggish	202	128 (63%)	64.44	18.74
Neutral	336	224 (67%)	57.71	21.95
Another emotion	1	1 (100%)	65.00	0
I don't know	80	63 (79%)	48.89	26.22

When analysing negative emotion intensity as a predictor of voice‐hearing, the concurrent and time‐lagged models, with and without the inclusion of Sluggish, showed no significant results (Table C and D in Appendix S1).

The intensity of positive emotions will predict the presence of voice‐hearing.

When analysing positive emotion intensity as a predictor of voice‐hearing (Table E in Appendix S1), the concurrent model was statistically significant (*p* < .001); however, the odds ratio confidence interval contained 1, and the standard error was 0, suggesting an unstable model. The time‐lagged model was not significant.

When emotion intensity ratings of Relaxed were removed from the positive emotion intensity total (Table F in Appendix S1), neither the concurrent model nor time‐lagged models were significant.

The intensity of stressful events will predict the presence of voice‐hearing.

Stressful events significantly predicted the presence of voices concurrently and at the next time point (see Table 3). With each one‐unit increase in stressful events (on a scale from 0 to 10), the odds of hearing voices (on a scale from 0 to 1) increased by 82%, in the moment.

**TABLE 3:** Stressful events on presence of voices.

Concurrent model	Model ICC (grouped by participant)					
Stressful event on presence of voices	.75					
	**Coefficient**	**OR**	***SE***	***p***	**95% CIs (bootstrapped)**	
					**Lower**	**Upper**
Stressful events	0.60*	1.82*	0.41	.008	1.13	3.54
**Time‐lagged model**	**Model ICC (grouped by participant)**					
Stressful event on presence of voices	.74					
	**Coefficient**	**OR**	***SE***	***p***	**95% CIs (bootstrapped)**	
					**Lower**	**Upper**
Stressful events	0.53*	1.71*	0.35	.009	1.13	2.97

* *p* < .010.

The time‐lagged model was also significant, showing that with each one‐unit increase in ratings of stressful events (on a scale from 0 to 10), the odds of voice‐hearing increased by 71% in the next time point.

Overall, more stressful events were related to significantly higher odds of hearing voices, both in the moment and at the next time point.

The intensity of pleasurable life events will predict the presence of voice‐hearing.

When analysing pleasurable event ratings as a predictor of voice‐hearing, the concurrent and time‐lagged models showed no significant results (Table G in Appendix S1).

This relationship between stressful events and voice‐hearing will be moderated by negative emotion intensity.

The moderation relationship of negative emotion intensity on stressful events and presence of voices was not significant, even after removing Sluggish from the negative emotion intensity total (Tables H and I in Appendix S1).

This relationship between pleasurable events and voice‐hearing will be moderated by positive emotion intensity.

The fixed effects interaction (in Table 4) between pleasant events and positive emotion intensity had a negative association with hearing voices in the next time point. For each one‐unit increase in both pleasant event and positive emotion intensity together, the odds of hearing voices decreased by 33% in the next time point. Therefore, the co‐occurrence of pleasant events and intense positive emotions decreased the odds of hearing voices in the next time point. When controlling for the interaction, both main effects of pleasant events and positive emotion intensity had significant positive associations with hearing voices in the next time point.

**TABLE 4:** Positive emotions intensity on pleasant events and presence of voices.

Concurrent model	Model ICC (grouped by participant)		Pseudo‐*R* ^2^ (fixed)		Pseudo‐*R* ^2^ (total)	
Positive emotions intensity on pleasant events and presence of voices^a^	.08		.07		.68	
**Fixed effects**	**Coefficient**	**OR**	***SE***	***p***	**95% CIs (bootstrapped)**	
					**Lower**	**Upper**
Pleasant events	2.23**	9.27**	0.01	<.001	3.99	33.60
Positive emotion intensity	2.90**	18.1**	0.01	<.001	8.65	63.50
Pleasant events × positive emotion intensity	−0.41**	0.67**	0.00	<.001	0.49	0.75

^a^ Optimiser bobyqa applied for valid convergence. Default optimiser was Nelder Mead.

** *p* < .001.

However, consistent with the H2 findings, the model was no longer significant when relaxed emotion intensity was removed from the calculation of positive emotion intensity (see Table 5).

**TABLE 5:** Positive emotions intensity (relaxed removed) on pleasant events and presence of voices.

Concurrent model	Model ICC (grouped by participant)					
Positive emotions intensity (relaxed removed) on pleasant events and presence of voices^a^	.17					
**Fixed effects**	**Coefficient**	**OR**	***SE***	***p***	**95% CIs (bootstrapped)**	
					**Lower**	**Upper**
Pleasant event	1.37	3.95	12.00	.795	2.03	11.60
Positive emotion intensity (relaxed removed)	1042	4.13	11.40	.653	1.96	13.10
Pleasant event × positive emotion intensity (relaxed removed)	−0.25	0.78	0.31	.607	0.62	0.86

^a^ Optimiser bobyqa applied for valid convergence. Default optimiser was Nelder Mead.

## DISCUSSION

The present study used an EMA approach to investigate the temporal relationships between the perception of daily life events (stress and pleasure), emotion valence and arousal, and voice‐hearing onset in a clinical sample of transdiagnostic voice‐hearers. The results of this study build on existing literature and provide further insight into the complex interplay between these factors, with implications for understanding and supporting voice‐hearers. Stressful, but not pleasurable, events were significant predictors of voice‐hearing, both concurrently and at the next time point. Unexpectedly and counter to our predictions, neither negative nor positive emotion intensity predicted voice‐hearing, nor did they moderate the relationship between voice‐hearing onset and stressful or pleasurable events, respectively. Taken together, we can consider several explanations of these findings.

Firstly, the results suggest an important role in stress predicting voice‐hearing in daily life, independent of the intensity of negative emotions experienced. As there was no significant relationship between pleasurable events and voices, this aligns with some models of voice‐hearing (e.g. “hypervigilance hallucinations” model; Dodgson & Gordon, 2009) that postulate stressful life events may lead to cognitive systems designed to detect threat and avoid false negatives. Therefore, voice‐hearers may interpret ambiguous conditions as threats, have low beliefs about their capacity to cope with these threats (Laloyaux et al., 2016; Mian et al., 2017), and have increased stress/trauma responses to these threats (e.g. dissociation; Perona‐Garcelan et al., 2011; Piesse et al., 2023). This is also aligned with a qualitative voice‐hearer report suggesting voices are counterproductive protective functions to cope with difficulty (Strachan et al., 2023). Furthermore, as the prevalence of post‐traumatic stress disorder (PTSD) and trauma exposure are common amongst voice‐hearers (Fowler et al., 2006; McCarthy‐Jones, 2011; Offen et al., 2003), it is possible that high stress ratings are reflecting environmental triggers of voice‐hearers' PTSD or other trauma symptomology at that time. Finally, stressful events may increase voice‐hearers' self‐consciousness (self‐focussed attention), which has been correlated with source monitoring deficits (e.g. increased external attribution; Laloyaux et al., 2022; Morrison, 2019; Perona‐Garcelan et al., 2011; Tully et al., 2017), and may decrease the ability to attend to and interpret internal thoughts as voices in the moment (Watson & Clark, 1994), thereby increasing the experience of voice‐hearing.

Secondly, alexithymia may explain the lack of significance for both positive and negative emotion intensity in this sample of voice‐hearers (Cusworth et al., 2024). Difficulty in recognising and processing internal emotional states, and/or rapid efforts to suppress these difficult emotional states, as well as an externally oriented view on stressful events, may disrupt the normal frequency of self‐reported emotions and voice‐hearing experiences (Herbert et al., 2011; O'Driscoll et al., 2014; Tracy & Shergill, 2013). For example, if participants were engaging in suppression as a regulation strategy, this may lead to lower emotion intensity scores reported, while also increasing the likelihood of voices (as suppressing thoughts or perceptions can trigger source monitoring errors; Jones & Fernyhough, 2006).

Thirdly, we did not find evidence of intense pleasurable events predicting voice‐hearing, aligning with prior findings (Cohen & Minor, 2010). Positive emotions were also not predictors of voice‐hearing, suggesting voices may be more likely to be triggered by aversive experiences. When ‘relaxed’ was included in the model, the model was significant, indicating voices appeared to be less likely to occur in the same time point; though confidence intervals indicated this model was unstable. When ‘relaxed’ was excluded, the findings were non‐significant, indicating the model's instability may be attributed to the high frequency of ‘relaxed’ compared to other emotions. Further, as ‘relaxed’ would be considered a ‘low arousal’ emotion even if high intensity was endorsed, it may be an indicator that low arousal emotion predicts less voice‐hearing (Heller, 1993; Watson & Tellegen, 1985). Further investigation is needed.

### Clinical implications

The findings emphasise the need for personalised interventions that consider individual differences in the perception of stress. Stress function may differ depending on the need for care of voice‐hearers, with non‐clinical (‘healthy’) voice‐hearers showing lower cortisol levels during laboratory tasks (Baumeister et al., 2021). Given the complex interplay between emotions, life events and voice‐hearing—and individual experiences of these—interventions should be tailored to the specific needs of each voice‐hearer. This may involve identifying individualised ‘stressful event’ triggers for voice‐hearing and developing coping strategies to manage reactions and experiences of those triggers. Our findings suggest interventions or accommodations to reduce the intensity of stressful events may be helpful (e.g. modifying environments to reduce sensory overwhelm; Hirano et al., 2010).

Targeting negative perceptions of contexts by targeting cognitive and attentional biases, and an externally oriented view, may also offer avenues for treatment. For example, mindfulness‐based interventions to reduce overall reactivity via improved emotional awareness and attentional control over emotional cues (Guendelman et al., 2017), trauma‐focussed interventions to reduce reactivity to stressful trauma memory triggers (e.g. imagery rescripting, Paulik et al., 2019; Strachan et al., 2024), and mentalisation interventions (Allen et al., 2008; Webb et al., 2012) or cognitive‐behavioural interventions for improving emotional awareness and developing coping strategies to manage responses to stress (e.g. Unified Protocol, Allen et al., 2008; Barlow et al., 2017; Webb et al., 2012). Finally, clinicians may consider the use of techniques that require less emotional awareness (e.g. behavioural strategies, medication; Maisey et al., 2022; Webb et al., 2012).

### Limitations and future directions

The findings should be considered in relation to the following limitations. First, the findings used a mobile phone‐based self‐report to measure emotion intensity. In our study, measurement intervals varied, including within‐day lags of up to 90 min and potential between‐day lags of up to 12 h, with additional variability introduced by missed time points. It is possible that the effects of negative emotion may have dissipated by the time the next notification period began and, therefore, may have been too transient to capture some of the nuances of timing. Future research could explore this relationship further by using alternative measures of emotion intensity and reactivity, such as physiological measures or behavioural observations. While increasing measurement frequency is discouraged due to the participant burden (Bell et al., 2023), larger sample sizes would enhance statistical power and allow for increased analysis of time point effects. Additionally, future research could examine the role of emotion regulation processes such as suppression on the impact of negative emotion intensity.

Second, the sample was recruited from a psychology clinic research registry, and predominantly a sample of clinical voice‐hearers. Future research would benefit from including a sample of non‐clinical (or “healthy”) voice‐hearers to better understand the relationship of stress to voice‐hearing onset.

Third, our sample was not controlled for other diagnoses, other experiences, or medications. It is possible that voice‐hearers and/or controls have other co‐occurring symptoms, or their medication may influence their experience of stress, pleasure, or emotion; therefore, the specificity of findings is not yet established.

Future research should delve deeper into the specificities surrounding stressful events associated with voice‐hearing, including the potential bi‐directionality of this relationship, the relationship between stress reactivity and trauma exposure, mechanisms by which stress and emotions influence voice‐hearing, and the effectiveness of interventions targeting these factors. It may also be valuable to explore the role of alexithymia and its relationship to emotion and coping in voice‐hearers. Additionally, exploring the role of other factors such as social support, coping strategies and cognitive biases in the relationship between emotions, events and voice‐hearing could also be helpful.

## CONCLUSION

While previous research and voice‐hearing models have emphasised the role of emotion processes in voice‐hearing, this is the first study to examine and compare perceptions of daily life events (both stressful and pleasurable) and emotion valence and intensity, and their associations with the experience of voice‐hearing. Clinically, our findings underscore the relevance of stress and a negative perception of externally oriented events, with further research needed to explore useful interventions for targeting these mechanisms.

## AUTHOR CONTRIBUTIONS

**Kelly Cusworth:** Conceptualization; investigation; writing – original draft; writing – review and editing; visualization; methodology; formal analysis; data curation. **Sharla Cartner:** Writing – review and editing; formal analysis; writing – original draft. **Georgie Paulik:** Conceptualization; resources; supervision; writing – review and editing; methodology. **Neil Thomas:** Conceptualization; formal analysis; writing – review and editing; supervision. **Guillermo Campitelli:** Writing – review and editing; conceptualization; supervision. **Danielle C. Mathersul:** Conceptualization; methodology; supervision; resources; writing – review and editing; investigation.

## CONFLICT OF INTEREST STATEMENT

Georgie Paulik receives income for published chapters and books on psychotic disorders, and for training postgraduate professionals to work with voice‐hearers.

## Supporting information


Appendix S1.

## Data Availability

The data that support the findings of this study are available on request from the corresponding author. The data are not publicly available due to privacy or ethical restrictions.

## Footnotes

^1^
Basic descriptive statistics of missing data were examined, showing a significant negative correlation between voice‐hearing frequency and missing data frequency (*r* = −0.63, *p* < .01), suggesting participants with higher voice‐hearing frequency tended to have fewer missing data. No significant correlation was found between missing data frequency and mean emotion intensity (*r* = −0.06, *p* = .72).
