# Supplementary material for: The associations of stress, pleasure and emotion to voice‐hearing: An ecological momentary assessment study
Source: Psychol Psychother. 2025 May 19;98(4):918–33. doi: 10.1111/papt.12598 (PMC12617511; doi:10.1111/papt.12598)
Supplement: Supplementary file 1 — Appendix S1. [file PAPT-98-918-s001.pdf]

The Associations of Stress, Pleasure, and Emotion to Voice-Hearing: An Ecological Momentary Assessment Study

Supplementary Tables & Figures

Table A

Fit Indices for Two & Three-Level Models Analyses, Concurrent

Model	AIC	BIC
Model 1 Positive Emotions and Negative Emotions on Presence of Voices		
Two-level Model	1225.3	1274.7
Three-level Model	1509.9	1592.1
Model 1 Positive Emotions and Negative Emotions (Sluggish Removed) on Presence of Voices		
Two-level Model	1210.1	1259.4
Three-level Model	1492.7	1574.9
Model 1 Positive Emotions (Relaxed Removed) and Negative Emotions on Presence of Voices		
Two-level Model	1232.8	1282.1
Three-level Model	1506.9	1589.2
Model 2 Negative Emotion Intensity on Presence of Voices		
Two-level Model	1232.6	1259.8
Three-level Model	1474.8	1518.3
Model 2 Negative Emotion Intensity (Sluggish Removed) on Presence of Voices		
Two-level Model	1160.5	1187.4
Three-level Model	1360.2	1403.2
Model 3 Stressful Event on Presence of Voices		
Two-level Model	1254.8	1282.2
Three-level Model	1491.5	1535.4
Model 5 Positive Emotions Intensity on Presence of Voices		
Two-level Model	1214.8	1241.9
Three-level Model	1437.7	1481.1
Model 5 Positive Emotions (Relaxed Removed) Intensity on Presence of Voices		
Two-level Model	1000.0	1026.0
Three-level Model	1175.7	1217.4
Model 6 Pleasant Events on Presence of Voices		
Two-level Model	1258.5	1285.9
Three-level Model	1501.6	1545.4

Note: AIC = Akaike Information Criterion; BIC = Bayesian Information Criterion.

Table B

Fit Indices for Two & Three-Level Models Analyses, Lagged

Model	AIC	BIC
Model 1 Positive Emotions and Negative Emotions on Presence of Voices		
Two-level Model	1305.2	1354.5
Three-level Model	1509.8	1592.0
Model 1 Positive Emotions and Negative Emotions (Sluggish Removed) on Presence of Voices		
Two-level Model	1306.2	1355.5
Three-level Model	1579.9	1662.1
Model 1 Positive Emotions (Relaxed Removed) and Negative Emotions on Presence of Voices		
Two-level Model	1303.5	1352.9
Three-level Model	1578.5	1660.8
Model 2 Negative Emotion Intensity on Presence of Voices		
Two-level Model	1271.2	1298.4
Three-level Model	1516.4	1559.9
Model 2 Negative Emotion Intensity (Sluggish Removed) on Presence of Voices		
Two-level Model	1193.1	1220.0
Three-level Model	1393.5	1436.6
Model 3 Stressful Event on Presence of Voices		
Two-level Model	1294.1	1321.5
Three-level Model	1540.0	1583.9
Model 5 Positive Emotions Intensity on Presence of Voices		
Two-level Model	1252.2	1279.4
Three-level Model	1483.6	1527.0
Model 5 Positive Emotions Intensity (Relaxed Removed) on Presence of Voices		
Two-level Model	1030.2	1056.3
Three-level Model	1229.9	1271.7
Model 6 Pleasant Events on Presence of Voices		
Two-level Model	1298.0	1325.4
Three-level Model	1548.7	1592.6

Figure A

Number of surveys per participant

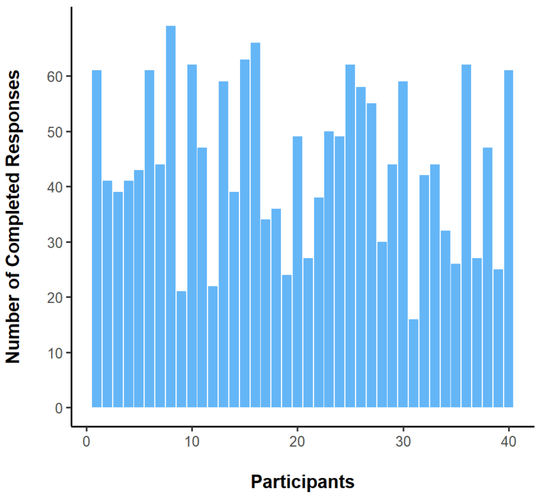

Figure B

Average number of surveys completed per day

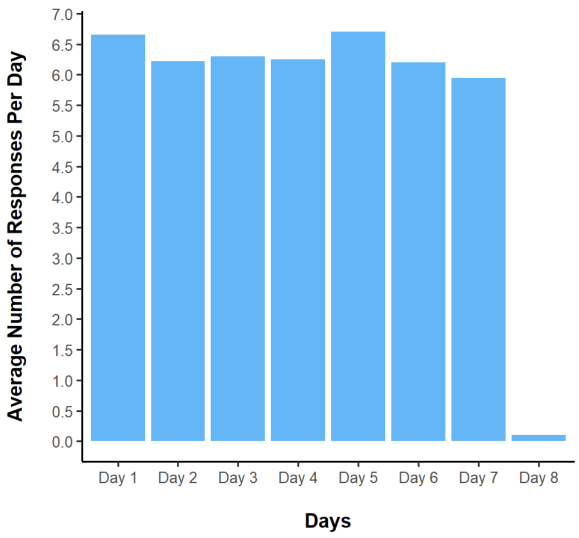

Table C

Non-significant concurrent and time-lagged models analysing Negative Emotion Intensity as a predictor of voice-hearing

Concurrent Model	Model ICC (Grouped by Participant)					
Negative Emotion Intensity on Presence of Voices	0.97					
	Coefficient	OR	SE	*p*	95% CIs (bootstrapped)	
					Lower	Upper
Negative Emotion Intensity	0.60	1.81	0.85	.206	0.71	5.26
Time-Lagged Model	Model ICC (Grouped by Participant)					
Negative Emotion Intensity on Presence of Voices	0.66					
	Coefficient	OR	SE	*p*	95% CIs (bootstrapped)	
					Lower	Upper
Negative Emotion Intensity	0.44	1.55	0.67	.311	0.61	4.22

Table D

Non-significant concurrent and time-lagged models analysing Negative Emotion Intensity (Sluggish Removed) as a predictor of voice-hearing

Concurrent Model	Model ICC (Grouped by Participant)		Pseudo-R^2 (fixed)		Pseudo-R^2 (total)	
Negative Emotion Intensity (Sluggish Removed) on Presence of Voices	.95		.06		.71	
	Coefficient	OR	SE	*p*	95% CIs (bootstrapped)	
					Lower	Upper
Negative Emotion Intensity (Sluggish Removed)	0.54	1.72	0.82	.250	0.67	5.22
Time-Lagged Model	Model ICC (Grouped by Participant)		Pseudo-R^2 (fixed)		Pseudo-R^2 (total)	
Negative Emotion Intensity (Sluggish Removed) on Presence of Voices	.98		.06		.79	
	Coefficient	OR	SE	*p*	95% CIs (bootstrapped)	
					Lower	Upper
Negative Emotion Intensity (Sluggish Removed)	0.62	1.86	0.88	.188	0.75	5.41

Table E

Non-significant concurrent and time-lagged models analysing Positive Emotion as a predictor of voice-hearing

Concurrent Model	Model ICC (Grouped by Participant)					
Positive Emotions Intensity on Presence of Voices	.98					
	Coefficient	OR	SE	*p*	95% CIs (bootstrapped)	
					Lower	Upper
Positive Emotions Intensity	-0.26**	0.77**	0.00	< .001	0.22	1.93
Time-Lagged Model	Model ICC (Grouped by Participant)					
Positive Emotions Intensity on Presence of Voices	.98					
	Coefficient	OR	SE	*p*	95% CIs (bootstrapped)	
					Lower	Upper
Positive Emotions Intensity	-0.48	0.64	0.34	.364	0.17	1.91

**$p < .001$.

Table F

Non-significant concurrent and time-lagged models analysing Positive Emotion Intensity (Relaxation Removed) as a predictor of voice-hearing

Concurrent Model	Model ICC (Grouped by Participant)					
Positive Emotions Intensity on Presence of Voices	.87					
	Coefficient	OR	SE	*p*	95% CIs (bootstrapped)	
					Lower	Upper
Positive Emotions Intensity	-0.42	0.65	0.26	.277	0.20	1.49
Time-Lagged Model	Model ICC (Grouped by Participant)					
Positive Emotions Intensity on Presence of Voices	.91					
	Coefficient	OR	SE	*p*	95% CIs (bootstrapped)	
					Lower	Upper
Positive Emotions Intensity	-0.43	0.65	0.24	.247	0.23	1.46

Table G

Non-significant concurrent and time-lagged models analysing Ratings of Event Pleasantness as a predictor of voice-hearing

Concurrent Model	Model ICC (Grouped by Participant)					
Pleasant Events on Presence of Voices	.98					
	Coefficient	OR	SE	*p*	95% CIs (bootstrapped)	
					Lower	Upper
Pleasant Events	-0.89	0.41	0.20	.063	0.10	1.01
Time-Lagged Model	Model ICC (Grouped by Participant)					
Pleasant Events on Presence of Voices	.97					
	Coefficient	OR	SE	*p*	95% CIs (bootstrapped)	
					Lower	Upper
Pleasant Events	-0.64	0.53	0.21	.103	0.19	1.20

Table H

Non-significant model analysing Negative Emotion Intensity as a moderator of the relationship between ratings of Event Stressfulness and voice-hearing

Concurrent Model	Model ICC (Grouped by Participant)					
Negative Emotion Intensity on Stressful Events and Presence of Voices[a]	.39					
	Coefficient	OR	SE	*p*	95% CIs (bootstrapped)	
					Lower	Upper
Stressful Event	0.06	1.06	1.63	.971	0.19	8.54
Negative Emotion Intensity	-0.48	0.62	0.47	.517	.37	1.23
Stressful Event x Negative Emotion Intensity	0.06	1.06	0.22	.780	.82	1.30

a. Optimiser bobyqa applied for valid convergence. Default optimiser was Nelder Mead

Table I

Non-significant model analysing Negative Emotion Intensity (Sluggish Removed) as a moderator of the relationship between ratings of Event Stressfulness and voice-hearing

Concurrent Model	Model ICC (Grouped by Participant)					
Negative Emotion Intensity (Sluggish Removed) on Stressful Events and Presence of Voices[a]	.37					
	Coefficient	OR	SE	*p*	95% CIs (bootstrapped)	
					Lower	Upper
Stressful Event	-1.44	0.24	0.37	.372	0.03	1.29
Negative Emotion Intensity	-0.69	0.51	0.33	.307	0.29	0.78
Stressful Event x Negative Emotion Intensity	0.25	1.28	0.27	.241	1.05	1.65

a. Optimiser bobyqa applied for valid convergence. Default optimiser was Nelder Mead
